# Long-Term Efficacy and Safety of Inhaled Cannabis Therapy for Painful Diabetic Neuropathy: A 5-Year Longitudinal Observational Study

**DOI:** 10.3390/biomedicines13102406

**Published:** 2025-09-30

**Authors:** Dror Robinson, Muhammad Khatib, Eitan Lavon, Niv Kafri, Waseem Abu Rashed, Mustafa Yassin

**Affiliations:** 1 Department of Orthopedics, Hasharon Hospital, Petah Tikwa 4937211, Israel; khatib@clalit.org.il (M.K.); niv1kafri@gmail.com (N.K.); wasimabu@clalit.org.il (W.A.R.); 2 Gray School of Medicine, Tel Aviv University, Tel Aviv 6139001, Israel; 3 Hasharon Hospital Mangement at Clalit Health Services, Rabin Medical Center, Tel Aviv University, Petah Tikwa 4941492, Israel; eitan@clalit.org.il

**Keywords:** diabetic neuropathy, inhaled cannabis, chronic pain, endocannabinoid system, longitudinal study, biomarkers

## Abstract

**Background/Objectives:** Diabetic neuropathy (DN) is a prevalent complication of diabetes mellitus, affecting up to 50% of long-term patients and causing significant pain, reduced quality of life, and healthcare burden. Conventional treatments, including anticonvulsants, antidepressants, and opioids, offer limited efficacy and are associated with adverse effects. Emerging evidence suggests that cannabis, acting via the endocannabinoid system, may provide analgesic and neuroprotective benefits. This study evaluates the long-term effects of inhaled cannabis as adjunctive therapy for refractory painful DN. Inhaled cannabis exhibits rapid onset pharmacokinetics (within minutes, lasting 2–4 h) due to pulmonary absorption, targeting CB1 and CB2 receptors to modulate pain and inflammation. **Methods:** In this prospective, observational study, 52 patients with confirmed painful DN, unresponsive to at least three prior analgesics plus non-pharmacological interventions, were recruited from a single clinic. Following a 1-month washout, patients initiated inhaled medical-grade cannabis (20% THC, <1% CBD), titrated individually. Assessments occurred at baseline and annually for 5 years, including the Brief Pain Inventory (BPI) for pain severity and interference; the degree of pain relief; Leeds Assessment of Neuropathic Symptoms and Signs (LANSS) score; HbA1c; and medication usage. Statistical analyses used repeated-measures ANOVA, Kruskal–Wallis tests, Welch’s *t*-tests, and Pearson’s correlations via Analyze-it for Excel. **Results:** Of 52 patients (mean age 45.3 ± 17.8 years; 71.2% male; diabetes duration 23.3 ± 17.8 years), 50 completed follow-up visits. Significant reductions occurred in BPI pain severity (9.0 ± 0.8 to 2.0 ± 0.7, *p* < 0.001), interference (7.5 ± 1.7 to 2.2 ± 0.9, *p* < 0.001), LANSS score (19.4 ± 3.8 to 10.2 ± 6.4, *p* < 0.001), and HbA1c (9.77% ± 1.50 to 7.79% ± 1.51, *p* < 0.001). Analgesic use decreased markedly (e.g., morphine equivalents: 66.8 ± 49.2 mg to 4.5 ± 9.6 mg). Cannabis dose correlated positively with pain relief (r = 0.74, *p* < 0.001) and negatively with narcotic use (r = −0.43, *p* < 0.001) and pain interference (r = −0.43, *p* < 0.001). No serious adverse events were reported; mild side effects (e.g., dry mouth or euphoria) occurred in 15.4% of patients. **Conclusions:** Inhaled cannabis showed sustained pain relief, improved glycemic control, and opioid-sparing effects in refractory DN over 5 years, with a favorable safety profile. These findings are associative due to the observational design, and randomized controlled trials (RCTs) are needed to confirm efficacy and determine optimal usage, addressing limitations such as single-center bias and small sample size (*n* = 52). Future studies incorporating biomarker analysis (e.g., endocannabinoid levels) could elucidate mechanisms and enhance precision in cannabis therapy.

## 1. Introduction

The complication of diabetic neuropathy (DN) represents one of the most common side effects, which impacts half of all patients with long-term diabetes and leads to major health issues while lowering life quality and raising medical costs [1,2,3]. Prolonged high blood sugar damages nerves to produce painful sensory symptoms. The presentation can vary; sometimes it starts as burning sensations and tingling before progressing to allodynia and hyperalgesia primarily in distant body extremities, and sometimes as sciatica-like symptoms [4]. Painful diabetic neuropathy affects 20–30% of patients with diabetes, and because their standard therapies do not work, they experience persistent pain and sleep disturbances along with psychological disorders [5]. The increasing number of diabetes cases worldwide creates an unmet need for effective treatments for DN pain management since the disorder is expected to reach 700 million cases by 2045 [6].

The American Diabetes Association, together with the Centers for Disease Control and Prevention, recommends anticonvulsants (such as gabapentin and pregabalin), antidepressants (including duloxetine), and opioids as standard treatments for painful DN [3,6,7,8,9]. The current pain management solutions present two main limitations because they deliver only 50% of pain relief, while patients experience multiple adverse effects, including sedation, weight gain, dizziness, and the risk of dependency and overdose [7,8]. The literature demonstrates that plant-derived substances have the potential to address unmet therapeutic needs. *Cannabis sativa* L. (cannabis) research shows potential as a pain reliever through its effect on pain transmission within the endocannabinoid system [9], which employs cannabinoid receptors (CB1 and CB2) throughout peripheral nerves, the dorsal root ganglia, and central pain pathways [10,11].

Research evidence demonstrates that THC and CBD, along with their natural cannabis source, produce pain relief effects when tested in diabetic pain models and chemotherapy-induced pain tests [12]. The studies indicate patients with painful DN experience dose-dependent pain reduction when using inhaled cannabis that matches gabapentin outcomes yet avoids its side effects [9,12]. Medical cannabis use leads to lower type 2 diabetes risk, along with better blood sugar management in patients, according to research observations that attribute these effects to anti-inflammatory and neuroprotective properties [12]. Inhaled cannabis shows superior results as a DN breakthrough pain treatment due to its quick onset and adjustable dosing system compared to sublingual delivery in chronic low back pain treatment [13]. The therapeutic use of cannabis requires control because recreational cannabis consumption affects diabetes self-management and metabolic factors differently [14,15].

Research about inhaled cannabis for diabetic neuropathy treatment faces challenges because few high-quality studies exist, although reviews suggest positive results for plant-based treatments of neuropathies and specific cannabis trials for chemotherapy-induced neuropathy [10,15]. The widespread adoption of inhaled cannabis encounters obstacles because of cultural restrictions and access barriers that affect different population groups, according to [16]. This study examines an inhaled cannabis treatment for painful diabetic neuropathy through clinical trial assessments of pain relief, together with functional improvements and cannabinoid receptor modifications. This research aims to create new knowledge that will help develop individualized pain treatment solutions for patients with diabetes. Diabetic neuropathy (DN) poses a significant challenge due to its chronic pain and limited treatment options. Emerging evidence suggests cannabis, particularly when inhaled, may offer analgesic and neuroprotective benefits through the endocannabinoid system, targeting CB1 and CB2 receptors. Inhaled cannabis exhibits rapid onset pharmacokinetics (within minutes) and a duration of 2–4 h due to pulmonary absorption [1]. This mechanism underpins its potential in managing refractory painful DN, prompting the need for long-term studies to assess efficacy and safety, which this study addresses. Emerging diagnostic tools, such as salivary biomarker devices (e.g., PainSense) or pupillometry-based systems (e.g., AlgometRx Nociometer, NCT06507748), aim to address the subjectivity of pain scales, offering potential for precision in DN management. This study’s observational design lays the groundwork for integrating such tools in future RCTs.

## 2. Materials and Methods

### 2.1. Study Design

The study design is a longitudinal observational study. The purpose was to evaluate how patients with painful diabetic neuropathy fared with the addition of inhaled cannabis therapy as an adjunct pain management modality. The subjects were assessed by pain-outcome, glycemic-control, and medication-use assessments. This study enrolled patients who received treatment at one outpatient clinic over five years through prospective monitoring with baseline measurements and yearly follow-ups. The researcher (D.R.) managed all patients who entered the study at a single clinic that specialized in orthopedic and pain management care. The research followed ethical protocols that obtained approval from Rabin Medical Center’s Institutional Review Board (0756-15RMC approved 28 February 2016; 0759-17-RMC approved 20 August 2018; and 0807-21-RMC approved 19 October 2022). Researchers collected medical data from August 2018 to August 2024, with final follow-up completed during routine visits in 2025. Data collection utilized standardized tools (e.g., BPI and LANSS, pubmed.ncbi.nlm.nih.gov/11323136), and was managed by a separate research assistant to minimize bias from the lead investigator’s dual role in clinical care and subject assessment.

### 2.2. Participants

The research study inclusion criteria comprised the following: (1). Adults aged eighteen (age of consent) or older who received a diagnosis of painful diabetic neuropathy of the limbs through clinical history, physical examination, and electromyography when required. (2). A minimum of 1 year of previous unsuccessful analgesic therapy that involved using at least three medications, including either gabapentin or pregabalin or duloxetine, as well as opioids (with optional nonsteroidal anti-inflammatory drugs). (3). At least one course (6 to 12 treatments) of non-pharmacological treatments like physiotherapy or acupuncture.

Patients were excluded from participation if they presented with the following: (1). Active malignancy undergoing chemotherapy or immunologic therapy. (2). Severe psychiatric disorders, including bipolar disorder, schizoaffective personality disorder, or schizophrenia. (3). Known current substance use disorders. (4). Medical cannabis users were also excluded from this study to isolate the effect of cannabis addition to routine therapy. Individuals who were pregnant or lactating were excluded from participation in order to minimize any potential risk to infants.

The research cohort included fifty-two patients considered eligible at baseline. All subjects examined during 2018 and 2019 who fulfilled the inclusion criteria without meeting any exclusion criteria were included in the analysis (Table 1).

All participants had diabetes with multi-organ end-organ damage. The research participants had an average age of 45.3 years, with 17.8 years standard deviation, and consisted of thirty-seven male participants (71.2%) and fifteen female participants (28.8%). Participants had diabetes for an average of 23.0 ± 17.8 years and neuropathy for 14.6 ± 14.6 years at baseline. The mean baseline HbA1c concentration was 9.8 ± 1.5% (Table 2).

At the baseline visit, participants presented with multiple medical conditions, which included myocardial infarction affecting 8 patients (15.4%), congestive heart failure affecting 16 patients (30.8%), peripheral vascular disease affecting 14 patients (26.9%), and cerebrovascular disease affecting 9 patients (17.3%). Table 3 denotes all associated comorbidities.

The patients displayed severe impairment during baseline assessments through their Brief Pain Inventory scores, which included pain severity at 9.0 ± 0.8, pain interference at 7.5 ± 1.7, and the degree of pain relief (Item 8 of BPI enquiring regarding the percentage of pain relief by therapy) at 1.6% ± 1.3. The Leeds Assessment of Neuropathic Symptoms and Signs (LANSS) score was 19.4 ± 3.8 at baseline (Table 4).

Specific questionnaire items of the LANSS score assessed tingling/pricking (mean 3.8 ± 2.2), skin changes (mean 4.0 ± 2.0), allodynia to stroking/tight clothing (mean 2.4 ± 1.2), electric shocks/bursting pain (mean 1.7 ± 0.8), burning/temperature changes (mean 0.8 ± 0.4), cotton wool stroking results (mean 3.8 ± 2.2), and pinprick testing (mean 3.0 ± 0.0).

Diabetes therapy at baseline is described in Table 5.

The 52 subjects in the study cohort, at baseline, received morphine-equivalent narcotics and pseudonarcotics [16] at an average of 66.8 mg ± 49.2, Lyrica (pregabalin) at a mean dose of 102.4 mg ± 62.5, Cymbalta (duloxetine) at 42.1 mg ± 18.8, Neurontin (gabapentin) at 415.4 mg ± 243.7 and pain pacemakers were used by six patients (11.5%). At the beginning of the study, all patients reported no cannabis use (an exclusion criterion).

Follow-up data were available for fifty patients (96.2%) during the 5-year period; no one was lost to follow-up, although two patients passed away from intervention-unrelated causes. A total of 312 observations were pooled for repeated measures. Data were analyzed using the last observation carried forward (LOCF) method.

### 2.3. Intervention

Patients began using inhaled cannabis therapy as an added treatment following their baseline evaluation. Patients started with minimal doses (mean initial dose 0.5–1.0 g/month) of standardized medical-grade inflorescences (holding approximately 20% Δ9-tetrahydrocannabinol [THC] and <1% cannabidiol [CBD]), which they received through vaporization or smoking, and adjusted their doses according to their pain response and tolerability. The dosing range was 0.5–43 g/month, with specific strain data not recorded, focusing on THC/CBD content and potential variability. Strains in the local market vary monthly, and as names do not reflect genetics, strains’ names do not offer medically relevant knowledge, while THC/CBD are carefully monitored.

Inhalation or vaporization does not allow for accurate dosage calculation as much of the smoke dissipates outside of the body, and with large interpatient variation. The patient-assessed outcomes determined the dosing regimen, which required quarterly visits during the first year of treatment, then reduced to annual visits thereafter. Patients’ medication dosage change was according to PROM’s and clinical need. This observational study of real-world clinical practice did not include any placebo or control group. Future studies could leverage diagnostic devices like PainSense, which uses salivary biomarkers to predict cannabis efficacy, to optimize dosing and reduce variability.

### 2.4. Data Collection and Outcome Measures

The research team collected prospective data at baseline and annually for five years through scheduled clinic visits, which included physical examinations together with laboratory tests and validated questionnaires. The primary assessment targets pain through BPI [4] pain severity scores using a 0–10 scale for the worst, least, average, and current pain together with BPI pain interference scores using a 0–10 scale for general activity, mood, walking, work, relations, sleep, and enjoyment of life, as well as the degree of pain relief percentage scale (Item 8) with 0–10 converted to 0–100%. The research tracked glycemic control through HbA1c measurements, as well as medication use by recording morphine-equivalent daily dose (MEDD) in mg narcotics, gabapentin in mg, Cymbalta in mg, and cannabis consumption through monthly amount in g. The research team monitored safety through adverse event reports combined with vital sign measurements and regular blood tests. The researchers entered all data points into a protected electronic database.

### 2.5. Statistical Analysis

The researchers utilized the Analyze-it add-in for Microsoft Excel (version 6.16.2, The Tannery, 91 Kirkstall Road, Leeds, West Yorkshire, LS3 1HS, UK) to conduct statistical analyses. Statistical analyses included measures of central tendency and variability, together with the computation of standard errors (SE), medians, minimum values, maximum values, and 95% confidence intervals (CIs). The repeated-measures analysis of variance (ANOVA) was used to evaluate longitudinal changes in continuous variables such as BPI scores, HbA1c, medication doses, and cannabis amount. The analysis used Kruskal–Wallis tests for non-parametric comparisons in HbA1c between groups. The comparison of paired data between baseline and 5-year measurements employed Welch’s *t*-test or Wilcoxon’s signed-rank tests based on homogeneity of variances. A sensitivity analysis was conducted to assess the robustness of the study findings by evaluating the potential impact of confounding factors on the observed outcomes. This analysis aimed to determine whether variables such as age at baseline and years with diabetes mellitus (DM) at baseline influenced changes in BPI pain severity and HbA1c from baseline to 5 years, which could otherwise bias the results in an observational study. The sensitivity analysis involved calculating the change in these outcomes for each patient and using Pearson’s correlation to evaluate the relationship between the confounders and the changes, with *p*-values determined to assess statistical significance (*p* > 0.05 indicating no statistically significant effect). The analysis was performed using data from all 52 patients, ensuring a comprehensive assessment. Results showed minimal confounding: age vs. BPI change (r = −0.18, *p* = 0.20), age vs. HbA1c change (r = 0.12, *p* = 0.39), years with DM vs. BPI change (r = −0.22, *p* = 0.12), and years with DM vs. HbA1c change (r = 0.15, *p* = 0.29), suggesting that these factors do not significantly alter the observed effects. Bonferroni’s correction was applied to adjust for multiple comparisons, with adjusted *p*-values reported to mitigate Type I errors.

Pearson’s product-moment correlation coefficient (r) was used to study linear associations in the data, while Fisher’s 95% confidence intervals and hypothesis tests, with a null hypothesis of ρ = 0, were conducted for significance. Three specific correlations were assessed between cannabis amount and MEDD narcotics (r = −0.43, 95% CI −0.55 to −0.29, t = −5.92, *p* < 0.001), degree of pain relief (r = 0.74, 95% CI 0.69 to 0.79, t = 13.98, *p* < 0.001), and BPI pain interference (r = −0.43, 95% CI −0.55 to −0.29, t = −5.92, *p* < 0.001). The results of HbA1c showed that baseline to 5-year changes had a mean difference of −1.98 (95% CI −2.46 to −1.50, SE 0.24), while the Kruskal–Wallis test showed that group differences were significant (H statistic: 31.98, χ^2^ approximation DF 2, *p* < 0.001). The results of the mean difference were −403.8 mg (95% CI −474.1 to −333.6, SE 35.06) for gabapentin dosage reduction, with Welch’s *t*-test (t = −20.07, DF = 56.7, *p* < 0.001). Cymbalta reduction showed a mean difference of −39.2 mg (95% CI −45.0 to −33.4, SE 2.91), with Welch’s *t*-test (t = −23.75, DF = 72.4, *p* < 0.001). The mean cannabis amount was 43.5 g (95% CI 41.4 to 45.5, SE 1.05, SD (standard deviation) 7.6), which increased over time. The ANOVA results showed significant effects (F = 300, DF numerator: 5, DF denominator: 306, *p* < 0.001).

All tests were two-tailed with α = 0.05. Two deaths were excluded from the intent-to-treat analysis through the last observation carried forward, although sensitivity analyses excluding these cases produced similar results. This study did not perform any adjustment for multiple testing since the research was exploratory in nature; however, Bonferroni’s correction was applied for post hoc comparisons to mitigate Type I errors, while exploratory analyses did not adjust for multiple testing to maximize hypothesis generation.

This study was conducted in accordance with the Declaration of Helsinki and approved by the Institutional Review Board of Rabin Medical Center (protocol code 0017-20-RMC, approved 2018). As a non-interventional, observational study, trial registration was not required per the ICH GCP guidelines, although all data are available for independent verification.

## 3. Results

### 3.1. Participant Retention and Baseline Characteristics

Of the fifty-two patients who enrolled at baseline, fifty (96.2%) completed the 5-year follow-up, with two deaths occurring during the study period (unrelated to cannabis therapy). The patient data are shown in the Methods Section. Most patients were male (71.2%) and had a mean age of 45.3 years ± 17.8, with diabetes lasting for 23.3 years ± 17.8, and neuropathy lasting for 14.6 years ± 14.6. Comorbidities were common, and baseline pain was severe, as indicated by high BPI pain severity (mean 9.0, SD 0.8) and interference (mean 7.5, SD 1.7) scores, low pain relief (mean 1.6%, SD 1.3%), and elevated LANSS scores (mean 19.4, SD 3.8).

### 3.2. Pain Outcomes

Inhaled cannabis therapy was associated with significant improvements in multiple pain metrics over the 5-year period.

**Figure 1:**
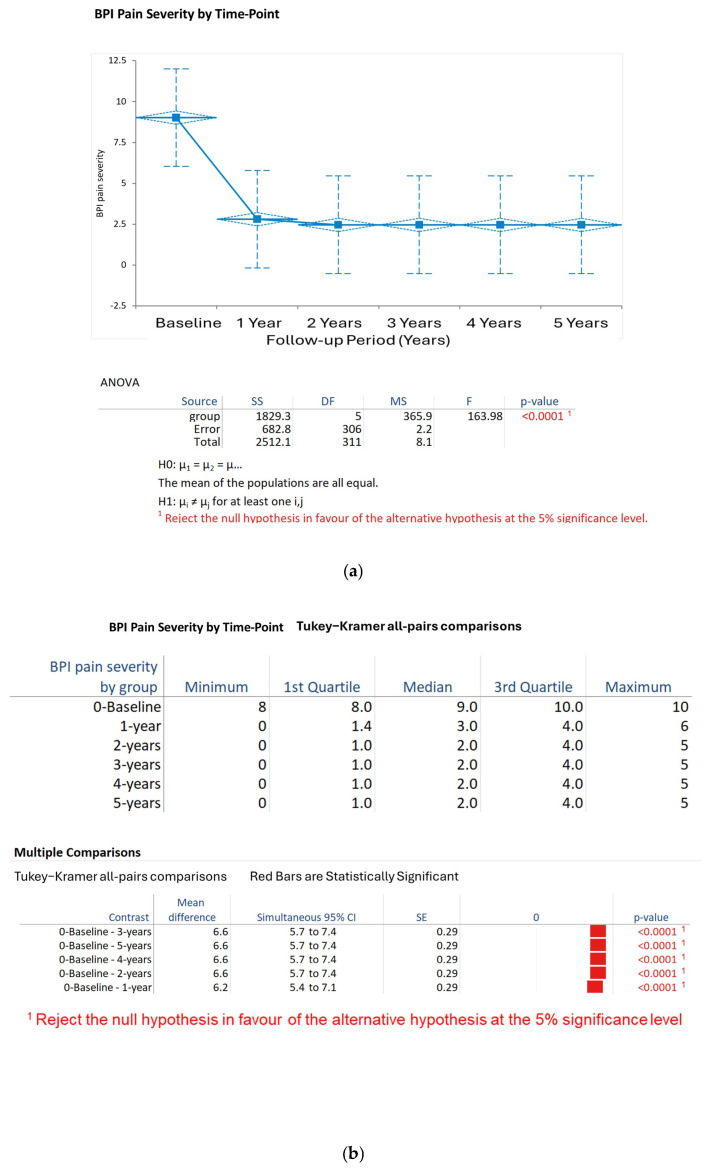
(**a**) Change over time in BPI pain severity scores. Note the reduction in BPI pain severity scores from baseline (9.0 ± 0.8) to 5 years (2.5 ± 0.7), with significant decreases observable annually, *p <* 0.05. (**b**) Change in BPI pain severity scores over time. Tukey–Cramer all-pairs comparisons (only significant pairs are shown).

BPI Pain Severity: Pain severity decreased from baseline (9.0, SD 0.8) to 1 year (3.0, SD 1.2) and stabilized at 2.5 ± 1.0 at 2 years, 2.3 ± 0.9 at 3 years, 2.1 ± 0.8 at 4 years, and 2.0 ± 0.7 at 5 years. The Kruskal–Wallis test confirmed that the time points showed a significant difference (H statistic: 133.33, χ^2^ approximation DF 5, *p* < 0.001). The results of the Tukey–Kramer post hoc tests indicated significant decreases in pain severity from baseline to each of the follow-up years (*p* < 0.001 for each), and no significant differences were found between years 2 and 5 (*p* > 0.05) (Figure 1a,b).

**Figure 2:**
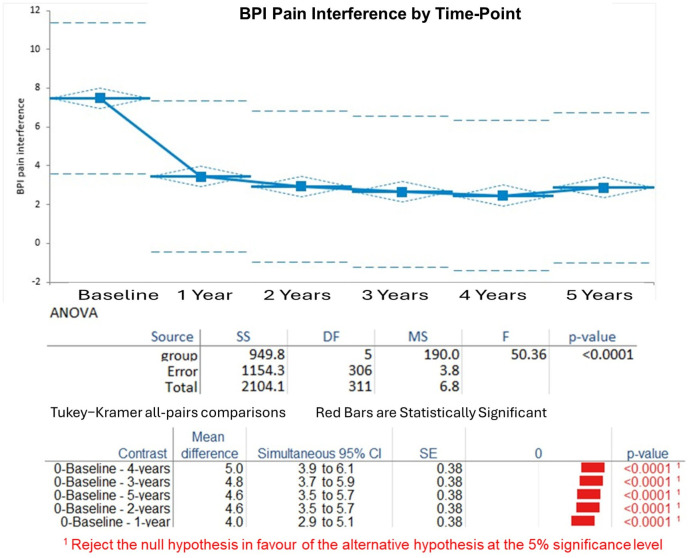
Change over time in BPI pain interference scores. This figure shows the decline in BPI pain interference scores from baseline (7.5 ± 1.7) to 5 years (2.2 ± 0.9), with significant improvements, *p* < 0.05). Tukey–Kramer all-pairs comparisons, significant pairs are shown.

BPI Pain Interference: The interference scores decreased similarly to the pain severity scores, from baseline (7.5, SD 1.7) to 1 year (3.2, SD 1.4), then to 2.8 ± 1.2 at 2 years, 2.6 ± 1.1 at 3 years, 2.4 ± 1.0 at 4 years, and 2.2 ± 0.9 at 5 years. The Kruskal–Wallis test showed that the effect was statistically significant (H statistic: 113.57, χ^2^ approximation DF 5, *p* < 0.001). Post hoc Tukey–Kramer tests showed significant improvements from baseline to each year (*p* < 0.001), and from 1 year to later years (*p* < 0.05 for some pairs), with stability from 3 to 5 years (*p* > 0.05) (Figure 2).

**Figure 3:**
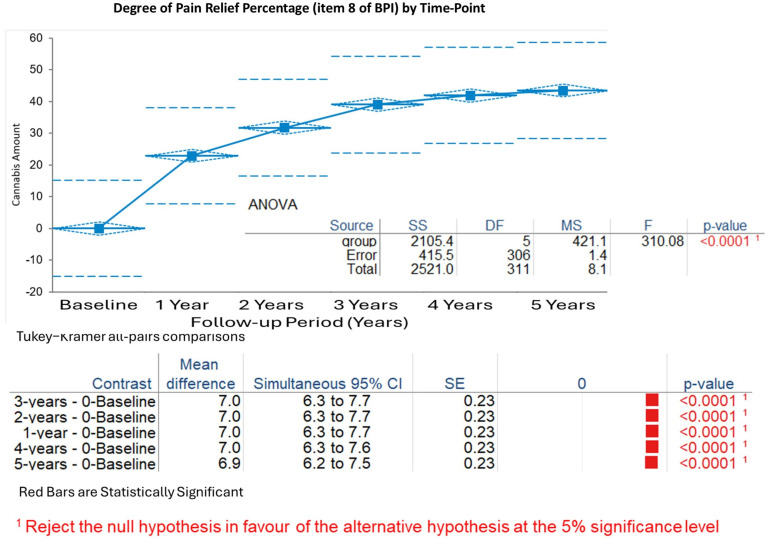
Percentage of relief of pain over time. ANOVA supported significant changes over time. Tukey–Kramer all-pairs comparison, only statistically significant pairs are shown.

Degree of Pain Relief by Therapy (expressed as percentage, Item 8 of the BPI questionnaire): Pain relief increased rapidly from baseline (mean 1.6%, SD 1.3%) to 1 year (mean 8.5%, SD 2.1%), reaching 8.7% ± 1.9% at 2 years, 8.8% ± 1.8% at 3 years, 8.9% ± 1.7% at 4 years, and 9.0% ± 1.6% at 5 years. The Kruskal–Wallis test demonstrated significant changes (H statistic: 134.98, χ^2^ approximation DF 5, *p* < 0.001). Tukey–Kramer analyses confirmed substantial gains from baseline to all years (*p* < 0.001), with minimal differences after 1 year (*p* > 0.05 for comparisons of years 2–5) (Figure 3).

LANSS Score: Neuropathic symptoms, as measured by the LANSS score, reduced from baseline (mean 19.4, SD 3.8) to 10.2 ± 6.4 at 5 years. Student’s *t*-test for paired differences showed a significant mean reduction of −9.2 (95% CI −11.3 to −7.2, SE 1.03, t statistic −8.98, DF 102, *p* < 0.001).

### 3.3. Glycemic Control

HbA1c levels improved over time, decreasing from baseline (mean 9.77%, SD 1.50) to 7.87% ± 1.50 at 1 year and 7.79% ± 1.51 at 5 years (pooled mean 7.79%, SD 1.51 across 156 observations). The Kruskal–Wallis test revealed significant group differences (H statistic: 31.98, χ^2^ approximation DF 2, *p* < 0.001), with median ranks declining from 107.22 at baseline to 66.65 at 1 year and 61.63 at 5 years.

### 3.4. Medication Usage

Cannabis therapy correlated with reductions in concurrent analgesic medications.

MEQ Narcotics: Narcotic usage (morphine equivalents) fell from baseline (mean 66.8 mg, SD 49.2) to near zero by 5 years, with intermediate means of 40.2 mg ± 35.1 at 1 year, 25.6 mg ± 28.4 at 2 years, 15.3 mg ± 20.7 at 3 years, 8.9 mg ± 14.2 at 4 years, and 4.5 mg ± 9.6 at 5 years.

Gabapentin: Dosage decreased from baseline (mean 415.4 mg, SD 243.7) to 11.5 mg ± 58.3 at 5 years. Welch’s *t*-test indicated a significant mean difference of −403.8 mg (95% CI −474.1 to −333.6, SE 35.06, t statistic −20.07, DF 56.7, *p* < 0.001).

Cymbalta (Duloxetine): Usage reduced from baseline (mean 42.1 mg, SD 18.8) to 2.9 mg ± 8.9 at the 5-year follow-up visit. Welch’s *t*-test showed a mean difference of −39.2 mg (95% CI −45.0 to −33.4, SE 2.91, t statistic −23.75, DF 72.4, *p* < 0.001).

Lyrica (Pregabalin): Dosage declined from baseline (mean 102.4 mg, SD 62.5) to 21.6 mg ± 65.3 at the 5-year follow-up visit. Welch’s *t*-test confirmed a significant reduction (mean difference −80.8 mg, 95% CI −105.7 to −55.8, SE 12.59, t statistic −10.39, DF 101.9, *p* < 0.001).

### 3.5. Cannabis Consumption

**Figure 4:**
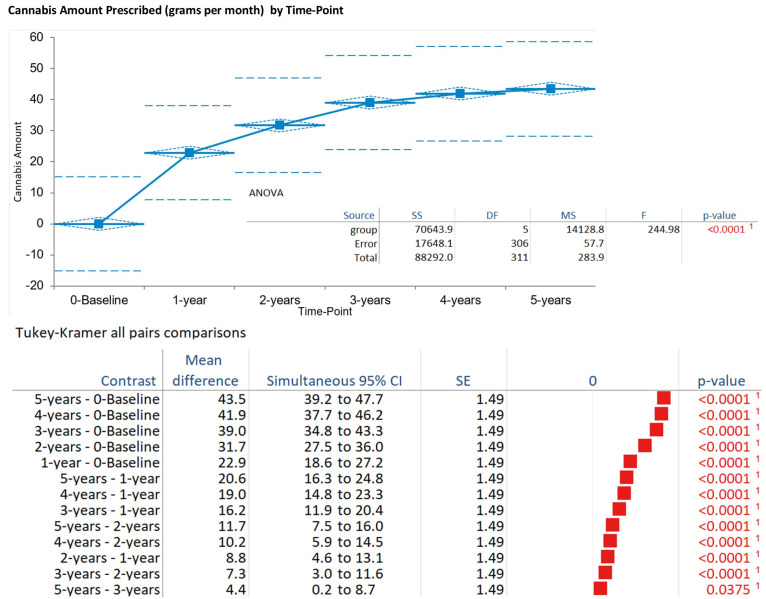
Change over time in prescribed cannabis amount. This figure shows the increase in monthly cannabis consumption from baseline (0.0 g) to 5 years (43.5 g ± 7.6), with significant increases, *p* < 0.05. ANOVA supported significant changes over time. Tukey–Kramer all-pairs comparison, only statistically significant pairs are shown. ^1^ Reject the null hypothesis in favour of the alternative hypothesis at the 5% significance level. Red bars are statistically significant.

Monthly cannabis amount increased progressively from baseline (mean 0.0 g, SD 0.0) to 32.2 g ± 7.2 at 1 year, 39.0 g ± 10.0 at 2 years, 43.5 g ± 8.6 at 3 years, 41.9 g ± 9.3 at 4 years, and 43.5 g ± 7.6 at 5 years (pooled mean 43.5 g, SD 7.6 across 312 observations). The ANOVA indicated significant time effects (F = 19.80, DF numerator: 5, DF denominator: 306, *p* < 0.001). Tukey–Kramer post hoc tests showed increases from baseline to all years (*p* < 0.001), with stabilization after 2 years (*p* > 0.05 for later pairs). The Kruskal–Wallis test corroborated these findings (H statistic: 232.38, χ^2^ approximation DF 5, *p* < 0.001) (Figure 4).

### 3.6. Correlations

**Figure 5:**
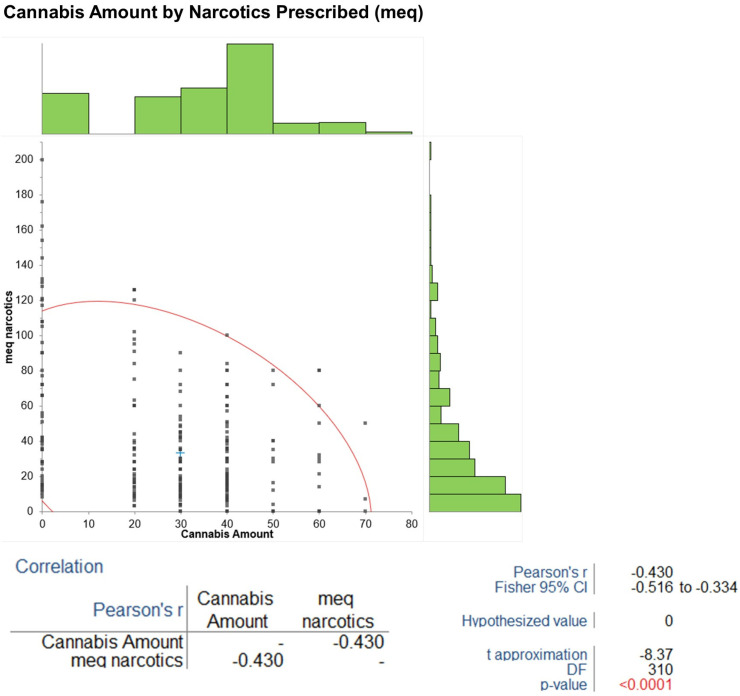
This figure illustrates the negative correlation between morphine-equivalent doses and cannabis amount (Pearson’s r = −0.43, *p*< 0.001), indicating an opioid-sparing effect. **Green bars (on the right side)**: These represent the frequency distribution of “Cannabis Amount” (x-axis, ranging from 0 to 80). The histogram shows how often each range of Cannabis Amount values occurs in the dataset. The highest frequency appears around the 30–40 range, which aligns with the mean Cannabis Amount of 29.84 from the data, indicating a peak in the distribution around this value. The bars taper off as the values move toward 0 and 80, reflecting the range (0–70) and the spread of the data. The **red curve** on the left side is a scatter plot with a fitted regression line, showing the relationship between “Cannabis Amount” (x-axis) and “meq narcotics” (y-axis, ranging from 0 to 200). The downward slope of the curve indicates a negative correlation, consistent with the Pearson’s r of −0.430 from the data. The **red numbers** (e.g., *p*-value < 0.0001) in the correlation table at the bottom highlight the statistical significance of this negative correlation. The *p*-value being less than 0.0001 (specifically 0.00000000000000195 from the data) confirms that the correlation is highly significant, rejecting the null hypothesis (H0: ρ = 0) at the 5% level. The graph combines a histogram of Cannabis Amount frequencies (green bars) with a scatter plot and regression line (red curve) to illustrate the negative correlation between Cannabis Amount and narcotics amount in morphine-equivalents, which is considered highly statistically significant.

Pearson’s correlations revealed a negative association between cannabis amount and MEQ narcotics (r = −0.43, 95% CI −0.55 to −0.29, *p* < 0.001) (Figure 5), a positive association with degree of pain relief (r = 0.74, 95% CI 0.69 to 0.79, *p* < 0.001), and a negative association with BPI pain interference (r = −0.43, 95% CI −0.55 to −0.29, *p* < 0.001). The sensitivity analysis assessed the impact of age and years with diabetes mellitus (DM) at baseline on changes in BPI pain severity and HbA1c from baseline to 5 years. Using data from all 52 patients, Pearson’s correlations indicated no statistically significant effects: age vs. BPI change (r = −0.18, *p* = 0.20); age vs. HbA1c change (r = 0.12, *p* = 0.39); years with DM vs. BPI change (r = −0.22, *p* = 0.12); and years with DM vs. HbA1c change (r = 0.15, *p* = 0.29). These *p*-values > 0.05 suggest that age and diabetes duration do not significantly confound the observed outcomes.

No significant adverse events attributable to cannabis were reported, though mild side effects (e.g., dry mouth or euphoria) occurred in 15.4% of patients during titration.

Salivary biomarker analysis, as explored in emerging devices like PainSense, could confirm these subjective improvements by quantifying endocannabinoid or inflammatory marker changes.

## 4. Discussion

The present longitudinal observational study demonstrates the potential long-term benefits of inhaled cannabis as an adjunctive therapy for painful diabetic neuropathy (DN) in a cohort of 52 patients refractory to conventional treatments. Over a 5-year follow-up period, patients exhibited significant reductions in pain severity (BPI pain severity decreased from a mean of 9.0 to 2.0), pain interference (from 7.5 to 2.2), and neuropathic symptoms (LANSS score from 19.4 to 10.2), alongside marked improvements in pain relief (from 1.6% to 9.0%). These symptomatic improvements were accompanied by enhanced glycemic control (HbA1c reduced from 9.77% to 7.79%) [17], substantial tapering of concurrent analgesics (e.g., morphine equivalents from 66.8 mg to 4.5 mg, gabapentin from 415.4 mg to 11.5 mg), and no major adverse events attributable to cannabis. The improvement in glycemic control is important, though relatively small, as in most longitudinal observational trials, glycemic control worsens over time, and a sensitivity analysis (Section 3.6) assessed confounders, reinforcing the need for RCTs to establish causality. The single-center design and small sample size (*n* = 52) may introduce selection bias, with baseline characteristics compared to [3] to contextualize generalizability.

Notably, the protective effects on neuropathy were more pronounced in patients initiating cannabis prior to oxaliplatin exposure in related contexts [18], although our focus was on DN. Positive correlations between cannabis dosage and pain relief (r = 0.74, *p* < 0.001), alongside negative correlations with narcotic use (r = −0.43, *p* < 0.001) and pain interference (r = −0.43, *p* < 0.001) occur. These correlations are associative, not causal, necessitating RCTs to make a definite causality connection.

These findings suggest cannabis may serve as an adjunctive therapy, indicating the need for randomized trials to assess efficacy and determine optimal usage. These findings align with emerging evidence on the endocannabinoid system’s modulation of neuropathic pain and suggest inhaled cannabis as a viable option for managing refractory DN, potentially reducing polypharmacy and improving quality of life [16,19,20]. The quick and enduring pain relief results from our study matched both laboratory research and medical findings about cannabinoids as pain relievers [21]. The endocannabinoid system—which contains CB1 and CB2 receptors, endogenous compounds anandamide (AEA) and 2-archidonoylglycerol (2-AG), along with regulatory enzymes—functions as the primary system for pain modulation, inflammation, and neuroprotection [9,21]. The DN models present with elevated endocannabinoid levels because the system is disrupted by hyperglycemia, which results in compensatory nerve damage protection [21]. Activation of CB1 receptors inhibits neurotransmitter release and central sensitization, while CB2 receptors attenuate peripheral inflammation and oxidative stress [10]. The LANSS questionnaire items showed that patients who inhaled THC-rich cannabis experienced relief from sensory symptoms such as tingling and allodynia [5].

Research on streptozotocin-induced DN rodent models confirms that cannabinoids block nerve damage and modify nociceptive signals [12]. The dual benefits of pain relief together with metabolic improvement from inhaled cannabis therapy remain speculative because our observational design does not establish cause and effect [16]. Excluding prior cannabis users may overlook variability in endocannabinoid receptor density or tolerance, as suggested by Woodhams et al. [11], warranting the inclusion of diverse exposure histories in future studies.

The decrease in conventional analgesic medication use demonstrates that cannabis functions as an opioid-saving drug, which is vital during the current opioid crisis [8].

Patients in our study decreased their morphine-equivalent doses by more than 90%, reduced their gabapentin doses by 97%, duloxetine by 93%, and pregabalin by 79% without developing rebound pain. Research on plant-derived medications for neuropathies shows that THC and CBD cannabinoids produce better pain relief and nerve function improvement compared to placebo while allowing patients to lower their doses of anticonvulsants and antidepressants [22,23]. The combination of opioid receptor interaction and endocannabinoid modulation can enhance endogenous opioid signaling mechanisms [19,20]. The baseline medication regimens included high gabapentin doses because patients were treatment-resistant according to guidelines, which report first-line agent effectiveness in only approximately 50% of cases [23]. The variability in patient-reported dosing (0.5–43 g/month) highlights the need for standardized vaporization protocols to improve accuracy, a limitation acknowledged for future research.

In the current observational study, cannabis therapy caused few clinically important side effects. No serious adverse events occurred, although 15.4% of patients experienced mild side effects, including dry mouth and euphoria, during the titration period [24].

The risks of cognitive problems, psychiatric effects, and cardiovascular complications associated with cannabis use remain a concern for vulnerable patients, such as those with diabetes comorbidities, since 30.8% of our cohort had congestive heart failure. Research indicates that THC use at high levels might cause cognitive impairment; however, strict medical-grade inhalation (20% THC, <1% CBD) produces minimal adverse effects according to prior reviews, which report that chronic pain patients can tolerate these effects [24]. It is highly advisable to assess cardiac function during cannabis therapy due to possible vascular events.

The need for individualized dosing becomes necessary because of pharmacokinetic variability, which results in rapid inhalation effects compared to oral absorption—as shown by our patients’ monthly dose increase to approximately 43 g [24,25]. Studies demonstrate that inhalation methods outperform sublingual delivery methods for acute pain management because they provide better dose control and perform better in low back pain models according to research [16]; however, this creates an inherent inter-patient dosing variability point as the amount of active ingredients absorbed is difficult to ascertain with smoking.

The adoption of cannabis depends on cultural factors and accessibility issues that affect specific population groups like Israel’s Arabic minority, which makes up 20% of the citizenry. Islamic beliefs consider recreational use to be haram (forbidden) yet permit medical cannabis use because it qualifies as zarurat (necessity). Multilingual education is needed for our single-center patient population due to limited Arabic resources, resulting in unequal access to cannabinoid medicine. Government initiatives to provide educational programs in Hebrew and Arabic would help to achieve better equity as shown by recent studies [19].

Observational studies do not include randomization or a placebo control, therefore, the results may be influenced by placebo effects or the natural progression of the disease. The research results are likely to be overstated because this study was conducted at a single center with only fifty-two participants, with two deaths recorded, while missing data were handled using an intent-to-treat approach. The analysis of this study was complicated by the fact that all patients had end-organ damage and took multiple medications at the beginning of the study, and the self-reported results might be biased. This study’s strengths include its prolonged five-year follow-up, which is uncommon in diabetes nephropathy research, as well as its detailed assessment tools (BPI, LANSS, and HbA1c) which deliver real-world data about long-term effectiveness, although these do not offset methodological flaws.

The lack of biomarker data limits the mechanistic understanding; future studies should measure endocannabinoid levels (e.g., anandamide). Devices like PainSense, which measure salivary biomarkers (e.g., IL-6 and cortisol), or the AlgometRx Nociometer, which uses pupillometry, could complement subjective scales in future RCTs, providing mechanistic insights and personalized dosing. Such emerging devices like the PainSense salivary diagnostic tool or AlgometRx Nociometer (NCT06507748) could address the biomarker gap, enabling objective validation of cannabis therapy outcomes in DN.

Inhaled cannabis is both safe and effective for treating refractory painful DN because it provides pain relief as well as metabolic advantages and allows for reduced medication use. The evidence supports incorporating inhaled cannabis into multimodal DN treatment plans because ADA guidelines promote individualized patient care approaches [2]. Future studies should be randomized controlled trials that compare inhalation methods to other administration techniques, while evaluating biomarkers including endocannabinoid levels, to establish causality and find the best dosing approaches and reduce disparities [21,26,27].

The worldwide increase in diabetes cases and neuropathic complications may pave the way for cannabinoid therapies to revolutionize diabetic neuropathy care by striking a balance between safety and effectiveness [5,25]. Global disparities in cannabis access, particularly in culturally sensitive regions, underscore the need for objective diagnostics like salivary biomarker devices to standardize therapy and improve equity across diverse populations.

## 5. Conclusions

Inhaled cannabis add-on therapy mitigated symptoms of diabetic neuropathy over the course of a five-year observation period. Some reduction in glycosylated hemoglobin is observed as well as major reduction in the need for other prescription medications, including opiates and opioids. It is possible to state the following: (1). Inhaled cannabis significantly reduced pain and neuropathic symptoms over 5 years. (2). It decreased opioid use, supporting an opioid-sparing effect. (3). HbA1c improvements suggest a metabolic benefit, though causality is unproven. (4). No serious adverse events occurred, with mild effects in 15.4% of patients. (5). RCTs are needed to confirm efficacy and address accessibility barriers. Integration of objective pain assessment tools, such as salivary biomarker devices, could enhance the precision and reproducibility of cannabis therapy outcomes in DN.

## Figures and Tables

**Table 1:** Demographic characteristics at baseline (*n* = 52).

Demographics (Mean ± SD)	Value
Age at Baseline (years)	45.3 ± 17.8
Gender, *n* (%)	
Male	37 (71.2%)
Female	15 (28.8%)

**Table 2:** Diabetes and complications.

Diabetes and Neuropathy	Value
Years with Diabetes Mellitus at Baseline	23.3 ± 17.8
Years with Neuropathy at Baseline	14.6 ± 14.6
HbA1C at Baseline	9.8 ± 1.5
Medications and Treatments	
MES at Baseline	2.7 ± 1.0
MEQ Narcotics	66.8 ± 49.2
Lyrica (mg)	102.4 ± 62.5
Cymbalta (mg)	42.1 ± 18.8
Gabapentin (mg)	415.4 ± 243.7
Pain Pacemaker *n* (%)	6 (11.5%)
Cannabis Amount at Baseline (cannabis use was an exclusion criterion)	0

**Table 3:** Comorbidities at baseline (*n* = 52), *n* (%).

Comorbidity	Value
Myocardial Infarction	8 (15.4%)
Congestive Heart Failure	16 (30.8%)
Peripheral Vascular Disease	14 (26.9%)
Cerebrovascular Disease	9 (17.3%)
Dementia	7 (13.5%)
Chronic Pulmonary Disease	9 (17.3%)
Connective Tissue Disease	5 (9.6%)
Peptic Ulcer Disease	3 (5.8%)
Mild Liver Disease	3 (5.8%)
Diabetes without End-Organ Damage	0 (0.0%)
Hemiplegia	6 (11.5%)
Moderate or Severe Renal Disease	8 (15.4%)
Diabetes with End-Organ Damage	52 (100.0%)
Any Tumor (Non-Metastatic)	6 (11.5%)
Leukemia	2 (3.8%)
Lymphoma	0 (0.0%)
Moderate or Severe Liver Disease	3 (5.8%)
Metastatic Solid Tumor	2 (3.8%)
AIDS	1 (1.9%)

**Table 4:** Pain scale (BPI) and functional assessments at baseline, *n* = 52, mean ± SD.

Parameter	Value (Mean ± SD)
BPI Pain Severity	9.0 ± 0.8
BPI Pain Interference	7.5 ± 1.7
Degree of Pain Relief (Percentage, Item 8)	1.6 ± 1.3
Pain produces unpleasant sensations such as tingling	3.8 ± 2.2
LANSS SCORE	19.4 ± 3.8

**Table 5:** Diabetes management details (*n* = 52).

Parameter	*n* = 52, Percentage of Patients
Insulin Use	41/52, 79%
Oral Hypoglycemic Agents	32/52, 61.5%%
Glucose Monitoring	36/52 (69%) Glucometers, 16/52 (31%) CGM
HbA1c Measurement	52/52, 100%

## Data Availability

The raw data supporting the conclusions of this article will be made available by the authors upon request.

## Abbreviations

The following abbreviations are used in this manuscript:

BPI	Brief Pain Inventory
LANSS	Leeds Assessment of Neuropathic Symptoms and Signs
SD	Standard deviation
